# T- and NK-cell populations with regulatory phenotype and markers of apoptosis in circulating lymphocytes of patients with CIN3 or microcarcinoma of the cervix: evidence for potential mechanisms of immune suppression

**DOI:** 10.1186/s13027-017-0166-1

**Published:** 2017-10-17

**Authors:** Olga V. Kurmyshkina, Pavel I. Kovchur, Ludmila V. Schegoleva, Tatyana O. Volkova

**Affiliations:** 1 Laboratory of Molecular Genetics of Innate Immunity, Institute of High-Tech Biomedicine, Petrozavodsk State University, Petrozavodsk, Russian Federation; 2 Department of Hospital Surgery, ENT Diseases, Ophthalmology, Dentistry, Oncology, Urology, Institute of Medicine, Petrozavodsk State University, Petrozavodsk, Russian Federation; 3 Department of Applied Mathematics and Cybernetics, Institute of Mathematics and Information Technologies, Petrozavodsk State University, Petrozavodsk, Russian Federation; 4 Department of Biomedical Chemistry, Immunology and Laboratory Diagnostics, Institute of Medicine, Petrozavodsk State University, Petrozavodsk, Russian Federation; 5 Institute of High-Tech Biomedicine, Petrozavodsk State University, Petrozavodsk, Russian Federation

**Keywords:** Immune suppression, Immunoregulatory mechanisms, Peripheral blood lymphocytes, Innate and acquired immunity, Apoptosis, Regulatory T cells, Natural killer cells, Cervical cancer, Preinvasive lesions

## Abstract

**Background:**

Processes and mechanisms responsible for systemic immune suppression in early-stage cervical cancer remain substantially underinvestigated. In this work, we focused on studying the frequencies of circulating regulatory T (CD4 and CD8 Tregs) and NK (NKregs) cells in parallel with assessment of apoptotic markers expression in T cells from patients with preinvasive and microinvasive cervical cancer, with the aim to determine whether up-regulation of apoptosis-associated markers in Т lymphocytes accompanies cervical cancer development and correlates with the change in percentages of regulatory cell populations at systemic level during the initial stages of invasive cervical cancer progression.

**Methods:**

Fourty two women with histologically confirmed cervical intraepithelial neoplasia grade 3 (CIN3, including carcinoma in situ) or cervical cancer (stage IA) and 30 healthy women (control) were enrolled in the study. Peripheral blood samples were taken immediately before surgery or any treatment and immediately subjected to multicolor flow cytometry.

**Results:**

Analysis of a combination of CD4/CD8, CD25, CD127, and FoxP3 markers revealed a statistically significant increase in the frequencies of Tregs within both the CD4 and CD8 subsets of circulating lymphocytes in patients with CIN3 and stage IA cancer. In contrast, lower numbers of NKregs (defined as CD16^dim/neg^CD56^bright^ subpopulation) and increased CD56^dim^/CD56^bright^ NK ratio were found in patients compared to controls, with the percentage of CD16^bright^CD56^dim^ cells (major subtype of circulating NKs) showing no difference. Patients also exhibited an increased expression of CD95 in total peripheral blood T lymphocytes, along with increased level of Annexin V binding to CD95-positive cells, suggesting higher susceptibility of T cells to apoptosis and potential involvement of CD95-dependent pathway in early-stage cervical cancer. Differential analysis of CD4 and CD8 T cells revealed different trends in the change of CD95 expression, confirming that this change likely has different functional significance for these two subsets. A search for correlations between the phenotypic parameters analyzed in this study was performed to demonstrate that women with early neoplastic lesions of the cervix, such as carcinoma in situ and microinvasive carcinoma, displayed a coordinated increase in expression of Treg markers in circulating lymphocytes, along with more pronounced cross-relationships between Treg numbers, CD95 expression on T cells, and apoptosis, compared to the control group.

**Conclusions:**

The results of this study suggest that a diversity of immune regulatory mechanisms that provide support for initial stages of invasive growth in cervical cancer patients includes systemic changes in the ratios between the principal regulatory and effector lymphocyte populations both within adaptive and innate immunity.

## Background

According to generally acknowledged conception, under the influence of cancer cells, innate mechanisms that maintain immune cell homeostasis and control the magnitude of immune responses can undergo dramatic dysregulation which leads to suppression of the immune system and enables the tumor to escape from immune attack and to progress. Regulatory pathways that mediate interactions between different functional types of immune cells during tumor development are currently under extensive investigation, and various in vitro and in vivo model systems for studying these interactions at the molecular and cellular levels are employed. Close attention is now being paid to lymphocytes that belong to adaptive or innate immunity and exhibit immunoregulatory properties; these lymphocytes include regulatory CD4/CD8 T cells (Treg), Th17/Th22 cells, γδT cells, regulatory B cells, regulatory NK cells (NKreg), NK-like T cells, and some other cell subsets. Significant progress has been made in the study of phenotypic characteristics of these diverse cell types and their impact on the activity of effector cells using experimental models. On the other hand, this progress highlights the need for additional studies to obtain evidence that carcinogenesis-related mechanisms mediated by these cells are physiologically relevant and can lead to dysregulation of immune homeostasis in cancer patients. Another, no less important issue is the ability of a tumor to exert not only local effects on distribution and activity of regulatory and effector cells throughout its entire development, but systemic effects as well, thus creating favorable conditions for growth both within the tumor microenvironment and at the level of “macroenvironment” [1]. Indeed, numerous studies have revealed dramatic changes in frequencies of the above-mentioned regulatory cells or other suppressor subsets (like M2-like macrophages and myeloid-derived suppressor cells, or MDSC) infiltrating tumor tissue. Altered expression of molecular markers indicating functional impairments in infiltrating cytotoxic cells has also been characterized in many cancer types, including those of epithelial origin. These data allow researchers to reconstruct local regulatory networks formed by immune cells and neoplastic cells at different stages of tumor progression. At the same time, there is a growing body of evidence showing alterations in the number and the phenotype of lymphocytes in blood, lymph nodes, and other metastatic sites in cancer patients, these findings demonstrating that the processes of systemically induced immune suppression can vary depending on tumor etiology, location, histologic type, and stage of development. Of particular interest are the changes in the immune system that occur in early phases of tumor progression, including precancerous lesions, because studying these changes provides an opportunity to evaluate the role of different immune cell populations in creating conditions for transition of premalignant lesions into invasive cancer. However, for many types of cancer such studies may be hampered by inability to diagnose precancerous conditions and early-stage cancer and, therefore, information on specific mechanisms maintaining immunodeficiency in patients is still lacking.

The cross-talk between Tregs and effector T cells has been recognized as a crucial factor in tumor development, with suppressive capacity of Tregs being realized in different ways, including inhibition of proliferation and activity of effector T cells, or induction of T cell apoptosis [2]. Cancer cells have the ability to promote expansion of CD4 Tregs population, which in turn, due to FasL/CD95L expression, are able to stimulate apoptosis in effector T lymphocytes via CD95-dependent pathway [2]. At the same time, Tregs are known to be more resistant to cell death because of elevated expression of endogenous anti-apoptotic molecules. Additionally, tumor cells and, as recently discovered, endothelial cells of tumor microvasculature can upregulate FasL/CD95L, contributing to higher apoptotic rates of activated CD95^+^ Т lymphocytes [3] and exhaustion of effector immune cells [4]. As illustrated by studies in patients with invasive hepatocellular carcinoma [5] and head and neck cancer [2], manifestations of this cross-talk can be detected in populations of peripheral blood lymphocytes, however, little is known about the extent they can reach during early stages of carcinogenesis. CD8 Tregs represent another cell type that has been recently recognized as the crucial component of the population of immunosuppressive Tregs. These cells were demonstrated to have similar capacity to suppress effector functions of T cells as CD4 Tregs, but in contrast to CD4 Tregs, their exact role in the regulation of antitumor immunity (including modulation of apoptosis) remains essentially unstudied. Lastly, the control over the number and the activity of various effector immune cells can be exercised by regulatory cells of innate immunity, as for example cytokine-producing NKregs, which involvement in pathogenesis of oncological diseases, especially at early stages, remains one of the least investigated issues [6, 7].

In light of the problem of immune regulatory mechanisms that maintain early stages of tumor progression, there is growing interest to the study of cervical cancer (CC). In its development, invasive carcinoma of the cervix goes through several morphologically distinct stages – cervical intraepithelial neoplasia (CIN) grade 1, 2, and 3 (including cancer in situ), and microinvasive cancer. The establishment of cervical neoplasia is mostly associated with high-risk papillomaviruses (HPV) that have evolved multiple strategies to evade and/or dysregulate host immunity mechanisms thus preventing clearance of infection [8]; in its turn, a host immune system failure to develop an effective response allows HPV to persist and increases the probability of malignant progression [9]. Pre-invasive and early-stage invasive cancers account for the largest number of cervical cancer cases, thus making possible comparative studies on immune parameters in the continuum of the cancer progression. A considerable amount of data has been amassed to date on molecular and cellular features altering tumor microenvironment upon CIN and СС development, such as dysregulated expression profile of cytokines or other immune-related markers, altered content and distribution of tumor-infiltrating lymphocytes, including cytotoxic cells and Tregs, with high-risk HPV considered to be a major driver of these events. In the case of invasive CC, alterations in the immune cell composition of tumor-positive and tumor-negative lymph nodes have also been reported, indicating early formation of a tolerogenic/immunosuppressive milieu (pre-metastatic niches) that precedes lymphatic spread of tumor cells [10–12]. Furthermore, there are some studies providing evidence that specific changes in immune parameters can occur in the systemic circulation of HPV-positive individuals and patients with CIN1–3 or CC. For example, peripheral blood T lymphocytes obtained from women with CIN1 were found to exhibit impaired ability to become activated in response to ex vivo stimulation with HPV antigens [13]. Several researchers have documented elevated frequency of circulating CD4 lymphocytes expressing Treg markers in blood samples of women with persistent HPV, CIN or CC [14–16]. Other studies have also identified that patients with CIN1/2 and CIN3 display altered numbers of some rare populations of circulating lymphocytes, which exert immunoregulatory/proinflammatory functions, such as Tc17 [17], Th17 and Th22 [18], NK-like CD8 cells [19], and CD4^+^NKG2D^+^ cells [20]. Deregulated expression of several activation markers and co-stimulatory/inhibitory molecules (such as NKG2D, CD28, and PD-1) was also described in peripheral blood lymphocytes from women with invasive cervical carcinoma or preinvasive lesions [21–23]. Additionally to immunoregulatory cell populations, some important changes in the levels of related cytokines (for example, IL-4, −6, −8, −10, −17, −22, −23, TNFα, IFNγ and other) have been revealed in the serum of CIN/CC patients [16, 18, 19], with TGFβ supposed to be one of the key factors operating the mechanisms by which regulatory lymphocytes are induced and can exert their suppressive effect [16, 23].

It is assumed that systemic abnormalities seen in patients with strictly local cervical lesions may arise as a consequence of persistent antigenic stimulation during chronic viral infection and facilitate neoplastic progression [22]; however there are still insufficient data to unequivocally confirm this assumption. Another question is that the observed shifts in the profile of regulatory and effector immune cells can be brought about by activation of apoptosis-related processes with engagement of Tregs as inducers of CD95-mediated lymphocyte death, but physiological significance of this relationship in cervical carcinogenesis is much less understood. In this work we focus on studying preinvasive and microinvasive cervical cancer as the earliest stages in disease development. We analyzed changes in the frequencies of peripheral blood CD4^+^/CD8^+^ regulatory cells and NKregs in parallel with assessment of TGFβ serum concentration, CD95 expression and the level of apoptosis in T cells, with the aim to determine: 1) whether up-regulation of apoptosis-associated markers in major Т-lymphocyte subsets accompanies CC development and correlates with the change in percentages of regulatory cell populations of adaptive and innate branches of immunity, and 2) whether these changes may extend to systemic level of immunity during the initial stages of invasive cervical cancer progression.

## Methods

### Patients and specimens

Samples of peripheral blood were obtained from 42 patients who underwent surgery in Oncological Dispensary of the Republic of Karelia: 27 women diagnosed with CIN3 (including cancer in situ, with average age at diagnosis 32.9 ± 7.4 years), and 15 with squamous cell carcinoma stage IА (CC stage IA, average age 31.3 ± 6.0) were examined. Stage of cancer was defined in accordance with the International Federation of Gynecology and Obstetrics (FIGO) system. CIN3 and CC IA diagnosis was based on comprehensive physical examination, extended colposcopy findings, cytology and histopathology tests, in full compliance with the approved standards for the diagnosis and treatment of patients with gynecological malignancies. All women engaged in this this study were informed and gave voluntary written consent. The research was approved by the Committee on Medical Ethics of Petrozavodsk State University and the Ministry of Healthcare and Social Development of the Republic of Karelia, and was done in accordance with the Declaration of Helsinki and good clinical practice guidelines. All women from patient group were positive for oncogenic HPV types (with the prevalence of HPV16 > 80%). Screening for the presence of HPV-DNA and identification of HPV-genotype were performed by polymerase chain reaction. Thirty healthy non-pregnant women (*n* = 14 of age 20–30, *n* = 11 of age 30–40, *n* = 5 of age 40–50) without cervical abnormalities and HPV-infection at the time of blood sampling served as normal controls. Venous blood was collected in a vacuum tube right before the surgery or any other treatment and immediately processed for multicolor flow cytometry. A portion of blood was centrifuged in order to obtain the plasma, which was divided into aliquots and frozen until processing by ELISA.

### Flow cytometry

To analyze lymphocyte surface expression of CD antigens, the following fluorophore-conjugated monoclonal antibodies were used: CD3-APC (Clone: UCHT1), CD4-FITC (Clone: MT310), CD8-FITC (Clone: DK25), CD16-FITC (Clone: DJ130c), CD95-RPE (Clone: DX2), CD56-RPE (Clone C5.9) (Dako, Austria), CD25-APC (Clone: 4E3), CD45-VioBlue (Clone: 5B1), CD127-RPE (Clone: MB15-18C9) (Miltenyi Biotec, Germany). To get rid of erythrocytes, 100 μl of the whole blood (per one probe) were lysed in ammonium chloride lysis buffer. The remaining cells were washed, resuspended in phosphate-buffered saline, counted, assessed for viability and further incubated with fluorochrome-labeled antibodies (1:20) for 30 min at room temperature in the dark. Optimal dilution for antibodies was determined empirically by titration. For blocking of non-specific antibody binding, FcR Blocking Reagent (Miltenyi Biotec) was added to cells in accordance with the manufacturer’s instructions. Total lymphocytes were gated according to FSC/SSC values and CD45 staining. For intracellular detection of FoxP3 transcription factor, cells were fixed and permeabilized using “FoxP3 Staining Buffer Set”, and stained with monoclonal anti-FoxP3 RPE-labeled antibodies (Clone: 3G3), all reagents from Miltenyi Biotec. Annexin-V-FITC kit (Miltenyi Biotec) was used to enumerate apoptotic lymphocytes. Cells were acquired on a MACSQuant Analyzer flow cytometer (Miltenyi Biotec) and analyzed using MACSQuantify software. Dead cells were excluded from analysis by propidium iodide staining and by forward/side scatter gating. The gains and compensation settings were adjusted, using single-stained cells. Not less than 100,000 cells were analyzed in each probe.

### ELISA

TGFβ1 serum concentration was measured using Platinum ELISA kit (eBioscience, USA), following the manufacturer’s instructions. The absorbance was read at 450 nm in SpectraMax i3 plate reader (Molecular Devices, USA).

### Statistical analysis

Data analysis was performed using R software [24]. Mann-Whitney U-test was used to evaluate the differences between the patient and the control groups; the difference was considered to be statistically significant at *p* < 0.05, *p* < 0.01, *p* < 0.001. The relationship between the parameters was investigated with the use of Pearson correlation coefficient. To describe a correlation pattern among the flow cytometric data, a weighted graph model was used, where the edges (arcs) received a weight corresponding to the correlation coefficient between two parameters; the two vertices (i.e. cell phenotypic parameters) were connected by an arc if the absolute value of the correlation coefficient was higher 0.6.

## Results

### Regulatory CD4/CD8 lymphocytes

**Fig. 1:**
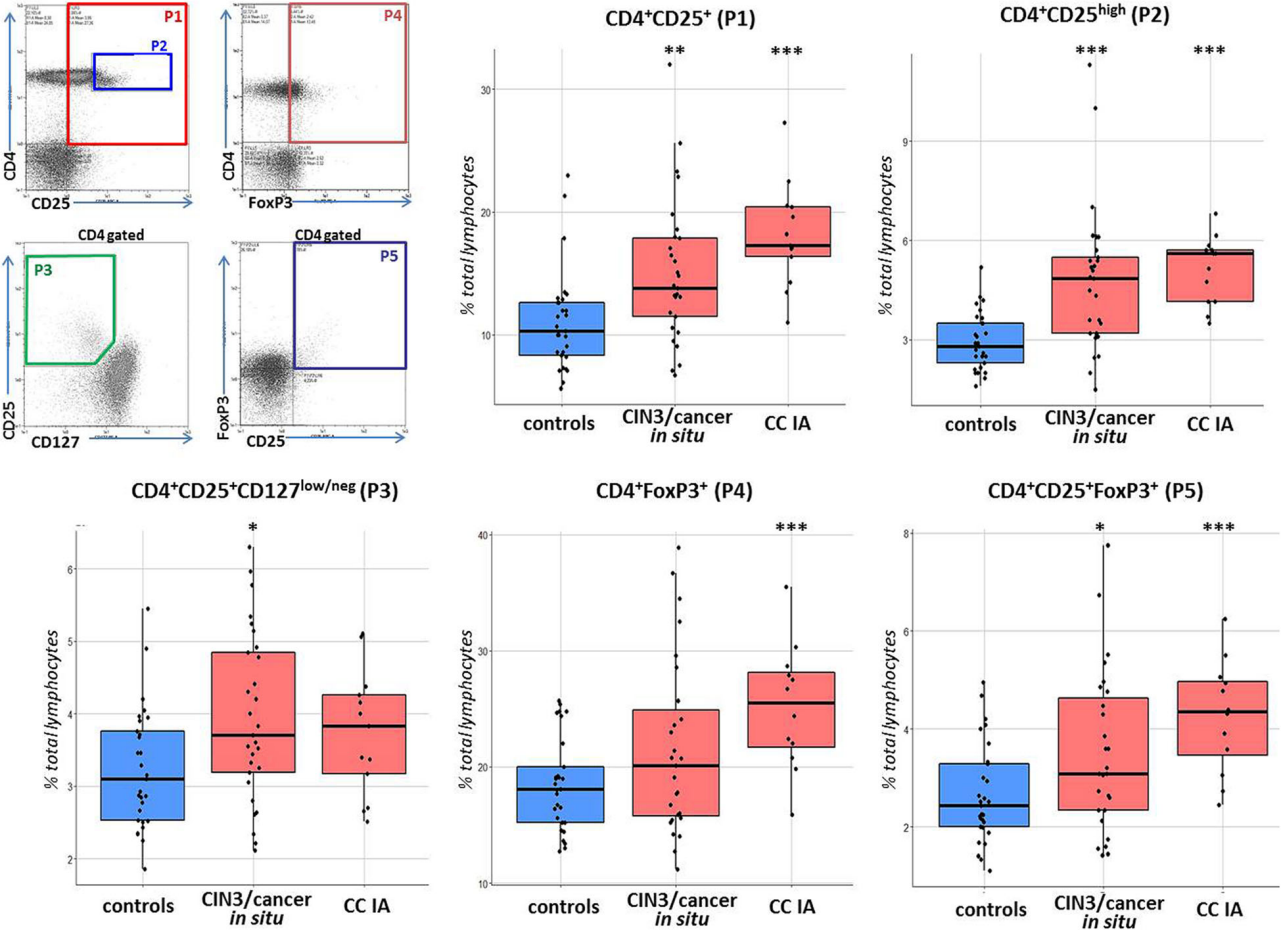
Boxplots representing the frequencies of cells with CD4 Treg-related phenotype in peripheral blood of patients and healthy donors. Lymphocytes were selected based on FSC/SSC distribution, gated for CD4 and further discriminated according to CD25 and CD127/FoxP3 staining (shown in the upper left). Here and below, individual values are shown as dots. The lower and upper hinges correspond to the first and third quartiles (the 25th and 75th percentiles). The middle line corresponds to the second quartiles (median, 50th percentile). The upper whisker extends from the hinge to the largest value no further than 1.5 * IQR from the hinge (where IQR is the inter-quartile range, or distance between the first and third quartiles). The lower whisker extends from the hinge to the smallest value at most 1.5 * IQR of the hinge. Data beyond the end of the whiskers are called “outlying” points and are plotted individually. Significant differences between the patients and the controls are designated by asterisks: * *p* < 0.05, ** *p* < 0.01, *** *p* < 0.001 (U-test)

**Fig. 2:**
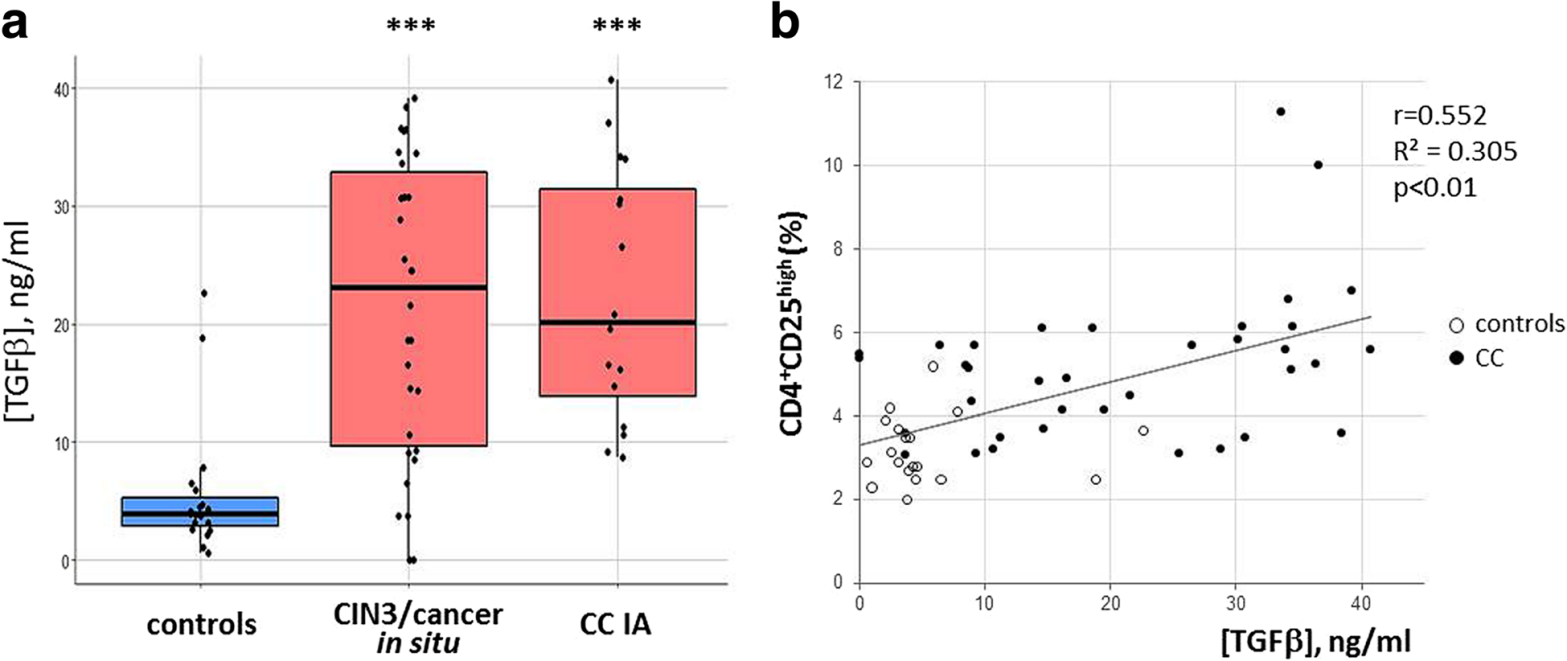
The change in TGFb1 levels in the sera of patients with cervical cancer compared to healthy individuals. **a** Boxplots showing increased concentration (ng/ml) of serum TGFβ1 in patients with pre- and microinvasive cancer; *** *p* < 0.001 (U-test). **b** Scatterplot illustrating the correlation between the level of TGFβ1 and the percentage of CD4^+^CD25^high^ lymphocytes in blood of cervical cancer patients (CC) and controls

The population of CD4 Tregs was determined by analyzing two combinations of essential Treg-markers: CD4^+^CD25^+/high^CD127^low/neg^ and CD4^+^CD25^+^FoxP3^+^ [25]. Total CD25^+^, FoxP3^+^, CD4^+^CD25^+^, CD4^+^CD25^high^, and CD4^+^FoxP3^+^ lymphocytes were also enumerated. No significant differences in the numbers of these cell populations were found between age subgroups of healthy controls. Patients with CIN3/cancer in situ and CC stage IA showed higher frequencies of CD4^+^CD25^+^ and CD4^+^CD25^high^ lymphocytes compared to the controls (Fig.1); the level of CD25 marker expression in total lymphocyte population was also increased in patients (*p* < 0.001, data not shown). Similarly, higher median levels of CD4^+^FoxP3^+^ cell numbers could be observed in patient groups than in the controls (with this result proved to be statistically significant for CC stage IA patients, *p* = 0.0006, Fig.1), and increase in the total number of FoxP3-expressing lymphocytes was also detected (*p* < 0.05, data not shown). The proportion of CD4^+^CD25^+^FoxP3^+^ cells among CD4-positive lymphocytes or total lymphocyte population was significantly elevated in peripheral blood samples from patients with CIN3/cancer in situ and CC stage IA (*p* = 0.037 and *p* = 0.0004, respectively), whereas the difference in the number of cells defined as CD4^+^CD25^+/high^CD127^low/neg^ lymphocytes between the groups of patients and controls appeared less pronounced. The percentage of CD4^+^CD25^+/high^CD127^low/neg^ lymphocytes was moderately correlated with the percentage of CD4^+^CD25^+^FoxP3^+^ lymphocytes (*r* = 0.604, *p* < 0.01), probably due to higher variability in CD127 expression in Tregs, as explained by Santegoets and colleagues who showed that FoxP3-expressing cells comprised approximately 70–85% of CD25^+^CD127^low^ population [25]. In addition to increased frequencies of peripheral CD4 lymphocytes with Treg-related phenotype, we revealed a higher concentration of serum TGFβ1 in patients with preinvasive and microinvasive CC (*p* < 0.001 for both patient groups, Fig.2a). Moderately strong positive relationship was found between serum TGFβ1 level and the percentage of CD4^+^CD25^high^ lymphocytes (*r* = 0.552, *p* < 0.001, Fig.2b).

**Fig. 3:**
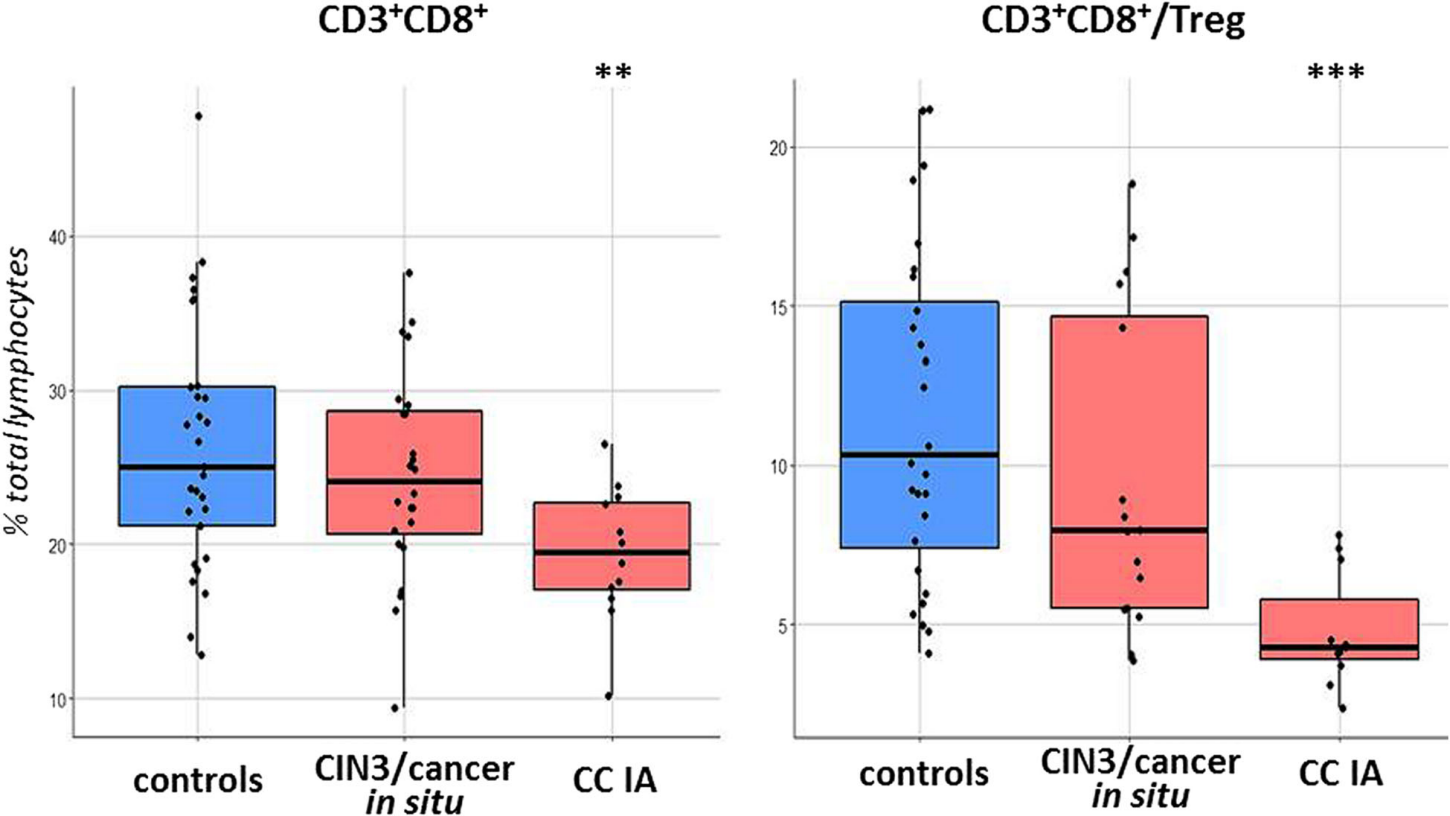
The change in CD3^+^CD8^+^ cell percentage and CD3^+^CD8^+^/Treg ratio of patients compared to healthy donors. ** *p* < 0.01, *** *p* < 0.001 (U-test)

The ratio of circulating CD8^+^ Т-cells to CD4^+^CD25^+^FoxP3^+^ regulatory lymphocytes (CD3^+^CD8^+^/Treg, Fig.3) was lowered in patients relative to the controls (*p* = 0.002 for the total patient group) as a result of increasing frequency of regulatory cells and declining numbers of CD3^+^CD8^+^ cells that could be seen upon invasive cancer formation (at the same time, there was no statistically significant difference in the number of CD3^+^CD4^+^ Т-cells between the patient and the control groups).

**Fig. 4:**
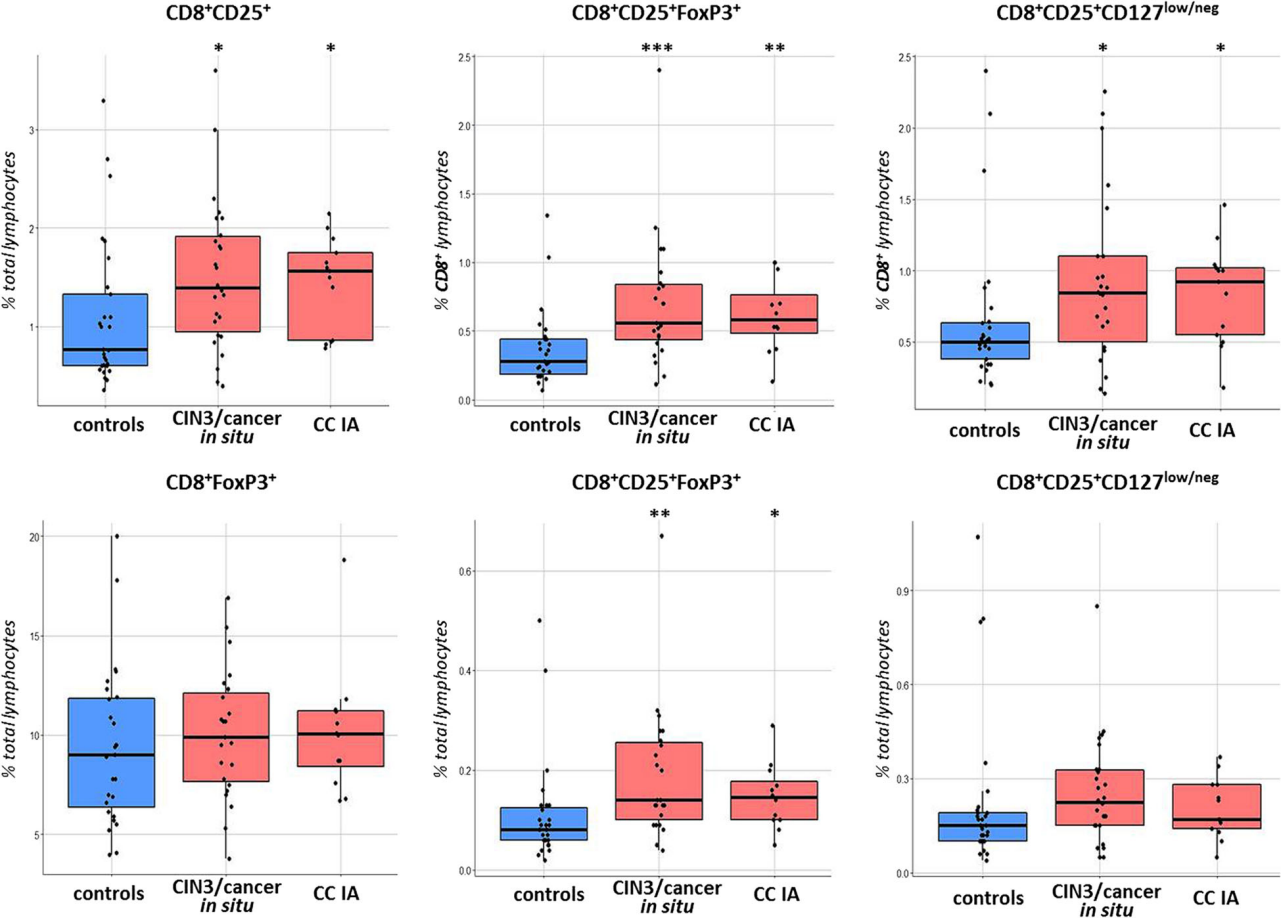
Boxplots showing the change in the frequencies of CD8 regulatory cell populations in peripheral blood of patients compared to healthy donors. The gating strategy was similar to that shown in Fig.1. * *p* < 0.05, ** *p* < 0.01, *** *p* < 0.001 (U-test)

Regarding CD8 Tregs, there is currently no consensus on a set of phenotypic markers that defines this population of lymphocytes possessing immunosuppressive properties. Nevertheless, it is known that, like CD4 Tregs, they are characterized by constitutive CD25 expression and, in contrast to effector CD8 T cells, they are positive for FoxP3. As shown in Fig.4, in blood samples from CIN3/cancer in situ/CC stage IA patients, the mean percentage of CD8^+^CD25^+^ lymphocytes was increased compared to control samples (*p* = 0.018); at the same time, a relatively wide range of values could be detected in healthy controls, which is in line with findings from others [26]. As confirmed by ex vivo testing, the CD8^+^CD25^+^FoxP3^+^ phenotype describes the population of CD8 cells with immunosuppressive capacity [26]. In our study, the frequency of CD25^+^FoxP3^+^ cells among circulating CD8^+^ lymphocytes was higher in CIN3/cancer in situ and CC stage IA samples compared to the controls (*p* = 0.0009 and p = 0,007, respectively, Fig.4). The average proportion of CD8^+^CD25^+^FoxP3^+^ cells in the whole lymphocyte population was also higher in cancer patients than in control subjects, but the difference was less pronounced (*p* = 0.002 for CIN3/cancer in situ and *p* = 0.017 for CC stage IA), presumably due to reduction in the level of CD8-bearing lymphocytes observed in women with cancer and low abundance of this cell population (0.1% on average) among peripheral blood lymphocytes from both patients and controls. In addition, it has been described that low level (or absence) of CD127 expression is characteristic of CD8 Tregs, therefore we analyzed CD8^+^CD25^+^CD127^low/neg^ lymphocytes and found their relative amount calculated as percentage of CD8^+^ lymphocytes was increased in patient-derived peripheral blood (similar to CD8^+^CD25^+^FoxP3^+^ counts) at *p* < 0.05, however, the change in the frequency of these cells expressed as a percentage of total lymphocytes was not statistically confirmed (Fig.4). Analysis revealed moderate correlation (*r* = 0.539, *p* < 0.001) between the percentages of CD4^+^ и CD8^+^ Tregs in patients (while these parameters were not correlated in healthy donor-derived blood samples, see Figs. 9 and 10), suggesting that coordinated expansion of immunosuppressive cell populations takes place upon disease progression. Taken together, these observations support the notion that early stages of CC development and progression (including, pre-invasive conditions) can be accompanied by a systemic increase in the frequencies of both CD4^+^ and CD8^+^ cells displaying Treg-associated phenotype.

### Expression of CD95 in CD4^+^ and CD8^+^ T cell populations

**Fig. 5:**
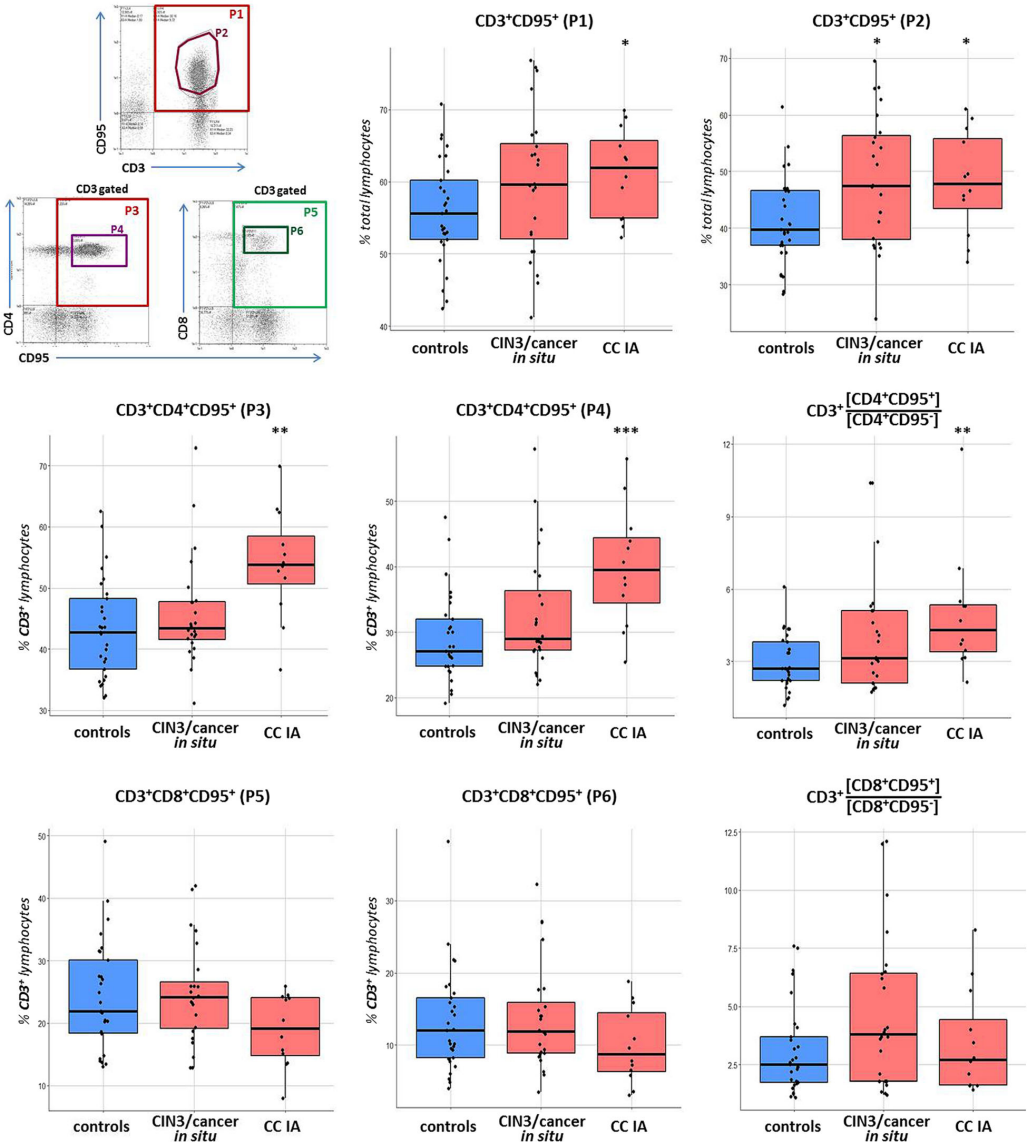
Boxplots summarizing flow cytometry data for the level of CD95 expression in populations of circulating Т lymphocytes in CIN3/CC women and in healthy controls. Lymphocyte gate was set according to FSC/SSC values; cells from a lymphocyte gate were then selected for CD3-positivity and analyzed for their CD4/CD8 and CD95 co-expression (shown in the upper left). Significant differences between the patients and the controls are designated by asterisks: ** *p* < 0.01, *** *p* < 0.001 (U-test)

CD95 membrane expression was estimated by flow cytometry both in total T-cell population and its CD4^+^/CD8^+^ subsets (Fig. 5). No significant differences in the level of CD95 expression on T cells, as well as in the number of CD4^+^ and CD8^+^ T cells could be detected between age subgroups within the control group. In patients, the amount of CD3^+^CD95^+^ lymphocytes (gate P1, Fig. 5) was increased as compared to the control group (*p* = 0.043), with this increase being more pronounced (*p* = 0.008) in a subpopulation of T cells expressing intermediate/high level of surface CD95 (gate P2). In general, the abundance of CD95^+^ cells among the total population of circulating lymphocytes was higher in patients with CIN3/cancer in situ and CC stage IA (*p* = 0.01, data not shown). Separate assessment of CD4^+^ and CD8^+^ T cells revealed different trends of CD95 expression during CC development: the proportion of CD4^+^CD95^+^ cells among T lymphocytes was higher in blood samples taken from patients (*p* < 0.05; gates P3, P4). On the contrary, a trend towards decreased proportion of CD8^+^CD95^+^ T cells was observed (*p* > 0.05) upon invasive cancer establishment (gates P5, P6). The number of CD95-negative cells was diminished within both CD4^+^ and CD8^+^ T cell populations of cancer patients, but in the case of CD8^+^ cells this reduction was more significant (p = 0.01, data not shown). Consequently, the ratio of CD95-positive T helpers to CD95-negative demonstrated significant increase upon disease progression (*p* = 0.005), whereas killer T cells exhibited no evident change in CD95^+^/CD95^−^ ratio (Fig. 5). Altogether, we can conclude that an increase in the frequency of circulating CD95^+^ Т cells seen in CC patients was associated primarily with the CD4^+^ T-cell population (with comparable, as mentioned above, frequencies of blood CD4^+^ T cells in CC patients and controls).

### The level of apoptosis in peripheral T lymphocytes

We further explored whether the expansion of suppressive Tregs, along with elevated levels of secreted TGFβ1 and CD95 death receptor surface expression, could be accompanied by enhanced level of T cell apoptosis in circulation of CIN/CC patients. The amount of apoptotic lymphocytes was evaluated by Annexin V (Anx) binding; dead cells and cellular debris were identified based on FSC/SSC parameters combined with propidium iodide (PI) staining, and were excluded from analysis. The quantity of Anx^+^PI^+^ lymphocytes (late apoptotic or necrotic cells) was relatively constant among peripheral blood samples from all groups examined (1.73 ± 0.20% for the control group and 1.93 ± 0.20% for the patient group, *p* > 0.05, data not shown), so these cells were also excluded.

**Fig. 6:**
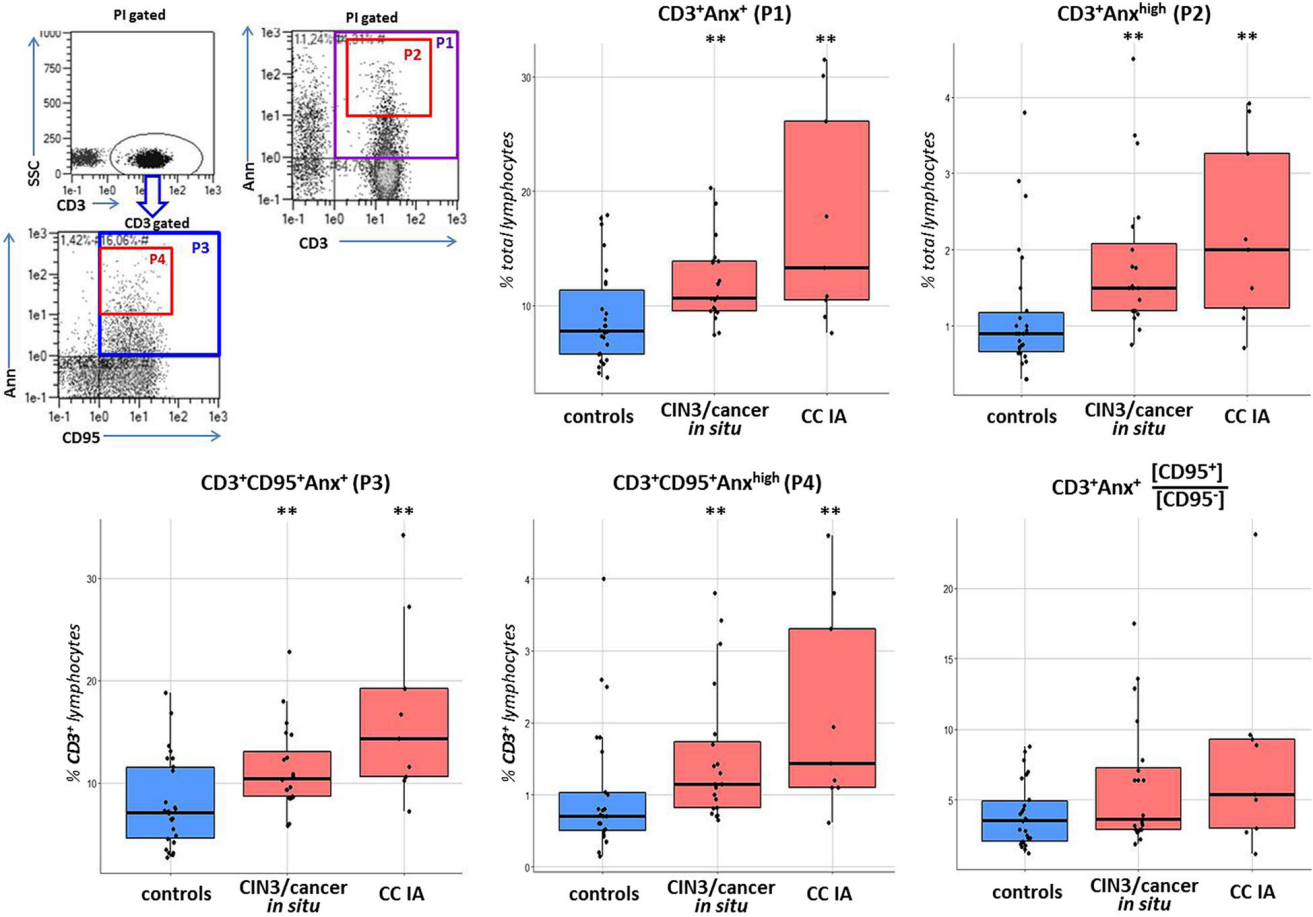
Boxplots displaying the level of apoptosis markers’ expression in the population of peripheral blood T lymphocytes from women with CIN3/CC and healthy donors. Live lymphocytes were gated by FSC/SSC and propidium iodid staining and then analyzed for their anti-CD3, anti-CD95 and Annexin V (Anx) binding. Among CD3^+^Anx^+^ cells (gates P1, P3), additional gates (P2, P4) were set to measure a subpopulation with high level of Annexin V binding. Significant differences between the patients and the controls are designated by asterisks: ** *p* < 0.01 (U-test)

As shown in Fig.6, the mean percentage of Т lymphocytes that bound Annexin V was higher in patients with CIN3/cancer in situ/ CC stage IA than in the controls at *p* < 0.01 (gates P1, P2). Further, by performing three-color staining, we assessed the abundance of CD3^+^Anx^+^ T cells co-expressing CD95 that could render them more susceptible to extrinsic/death receptor-mediated apoptosis. Indeed, there was a statistically significant increase in the proportion of CD3^+^Anx^+^CD95^+^ cells among CD3^+^ T lymphocytes in blood samples obtained from CIN/CC patients relative to the control samples (*p* < 0.01, gates P3, P4, Fig.6). In comparison, the amount of CD3^+^Anx^+^ cells, negative for CD95 expression, constituted a much smaller proportion of T lymphocytes (around 4 times less than the amount of CD3^+^Anx^+^CD95^+^ cells, in both patients and normal controls) and showed no significant difference between the groups examined (2.26 ± 0.28% for healthy controls and 3.00 ± 0.58% for patients, *p* > 0.05, data not shown). Consequently, the prevalence of CD3^+^Anx^+^CD95^+^ cells in population of Т lymphocytes calculated as CD3^+^Anx^+^[CD95^+^/CD95^−^] ratio (Fig.6) was higher in peripheral blood of patients (*p* = 0.051); furthermore, the percent of CD3^+^Anx^+^CD95^+^ cells appeared to be strongly correlated with the percent of CD3^+^Anx^+^ cells (*r* = 0.937, *p* < 0.001), suggesting growing importance of CD95-dependent pathway in apoptosis of peripheral Т lymphocytes during the development of invasive CC. As for CD3^+^Anx^neg^CD95^+^ cells, which constitute a major population of T lymphocytes and can be defined as activated effector T cells, the difference between healthy donors and CIN/CC patients was not statistically significant, despite some weak trend towards higher percentage of these cells in patients (55.2 ± 2.5% and 51.8 ± 2.0% of T cells in patients and controls, respectively; data not shown). Together, these findings support an assumption that the development of invasive CC may be associated not only with up-regulation of CD95 expression in peripheral blood lymphocytes, but rather with exacerbation of apoptosis-related processes in CD95^+^ T cells. These results also agree with our previous study demonstrated enhanced activity of the main CD95-regulated caspases (specifically, caspases −8 and −3) in total fraction of peripheral blood mononuclear cells at such early stages of CC progression as intraepithelial and microinvasive carcinoma [27].

**Fig. 7:**
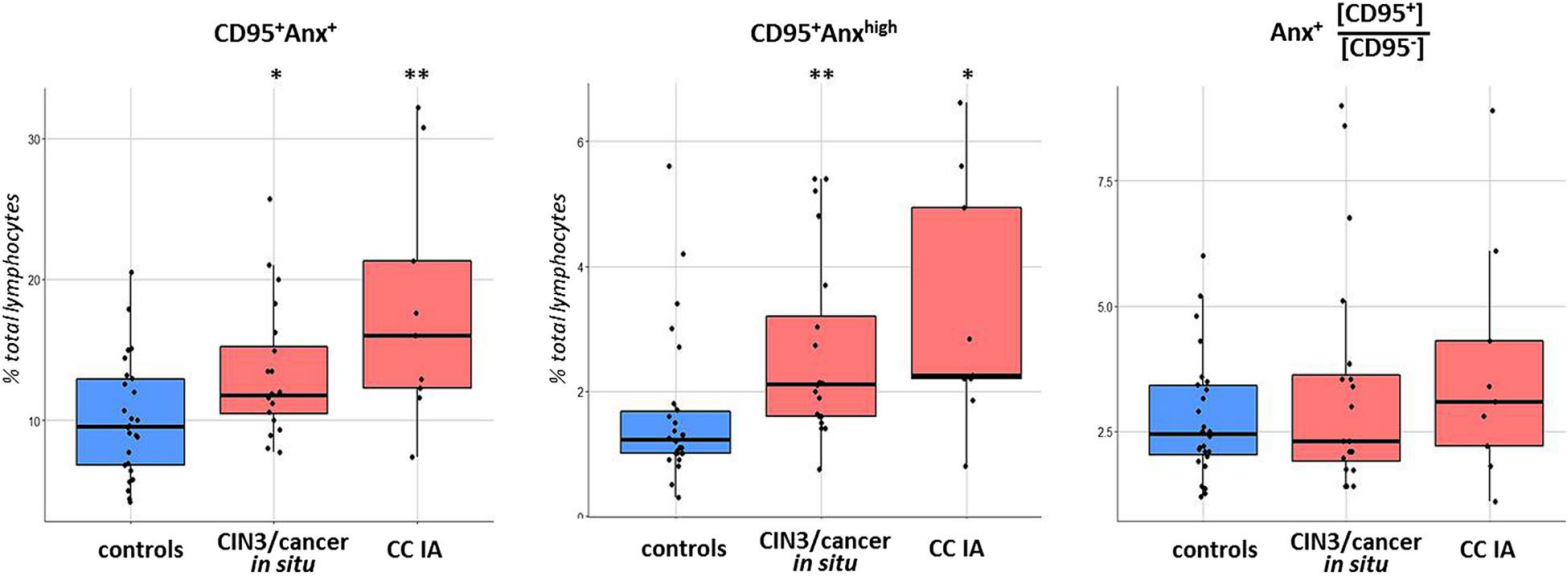
Boxplots representing the change of apoptosis markers’ expression in total population of peripheral blood lymphocytes from patients with CIN3/CC and control subjects (according to flow cytometry data). * *p* < 0.05, ** *p* < 0.01 (U-test)

In an assessment of the entire population of blood lymphocytes, an increase in the number of CD95^+^ cells co-stained with Annexin V was also noticed in cancer patients (*p* < 0.05; Fig. 7). This increase might be due in part to various populations of non-T lymphocytes (for example, B cells and natural killer cells) as we detected higher percent of CD3^−^Anx^+^ cells in blood from patients (6.74 ± 0.54% of lymphocytes) compared to normal controls (5.23 ± 0.43%, p < 0.05). However, when assessing the total lymphocyte fraction, the difference in the ratio of %Anx^+^CD95^+^ to %Anx^+^CD95^−^ cells between patients and controls was not found to be statistically significant (Fig. 7). These data seem to indicate, again, the specificity of alterations in apoptotic processes triggered by external signals (such as CD95L) with respect to T cell population during CC development, although the possibility that activation of extrinsic apoptotic pathways (including CD95-mediated) occur in other types of lymphocytes should not be discarded. Further studies are needed to corroborate this notion; in addition, the above observations of different pattern of changes in CD95 expression in the two major T cell subsets (CD4^+^ and CD8^+^) may imply different intensity of apoptotic processes, an issue which is also to be further investigated using modified flow cytometry staining/gating strategy.

### The innate arm of immunity: Regulatory NK lymphocytes (NKregs)

**Fig. 8:**
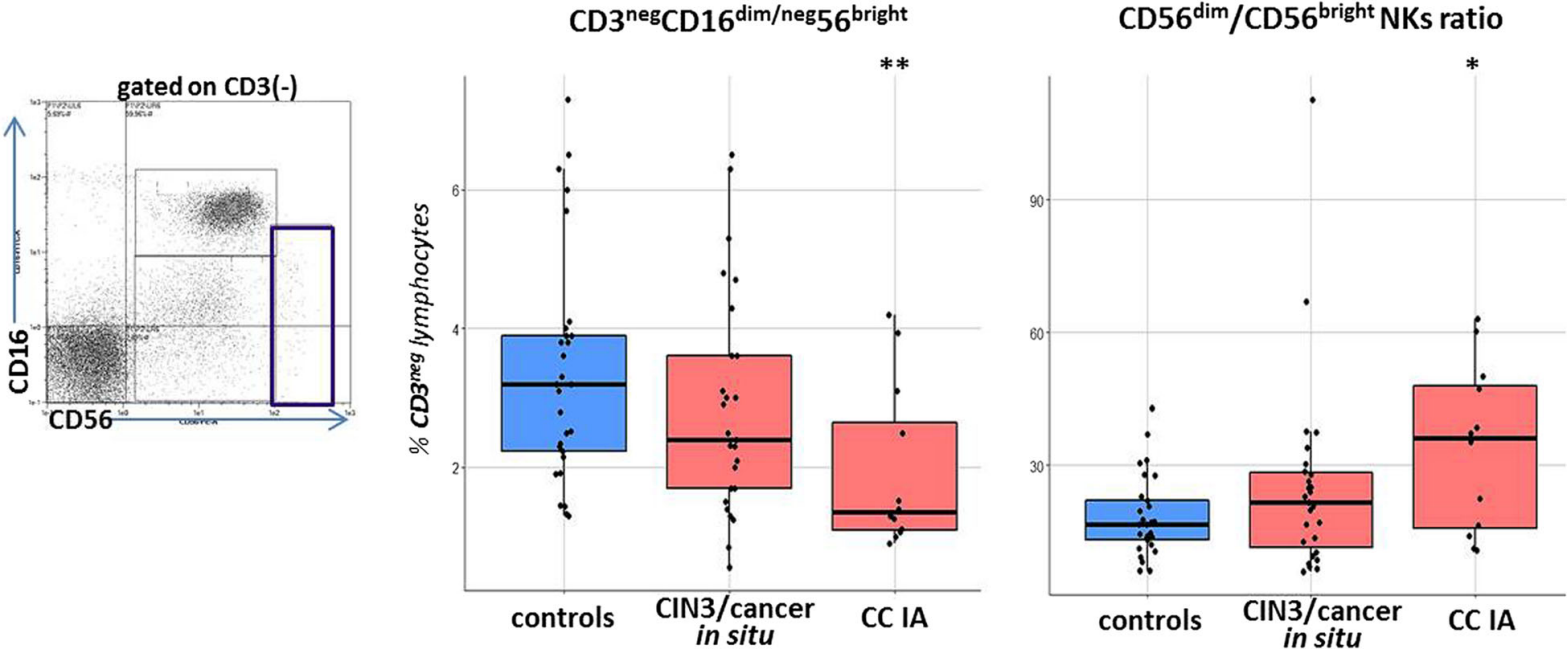
Percentage of peripheral blood CD56^bright^ NK cells (NKregs) and CD56^dim^/CD56^bright^ ratio within circulating NK population in different study groups. Lymphocytes were gated for CD3-negativity (a diagram on the left) and a population of interest was defined according to CD16/CD56 membrane expression levels (the gate for NKregs is depicted by a thick line). Significant differences between the patients and the controls are designated by asterisks: * *p* < 0.05, ** *p* < 0.01

NKregs have been described as a minor subpopulation of circulating natural killer cells having CD3^neg^CD16^dim/neg^CD56^bright^ phenotype. Compared to CD16^+^CD56^dim^ NK cells which represent the majority of NKs in peripheral blood, they are associated with relatively low cytolytic capacity (in the absence of appropriate activating signals), but are known for their high ability to produce cytokines and chemokines (including IFNγ, TNFα, GM-CSF, IL-10, IL-13, CCL3, CCL4), and therefore they are considered as immunoregulatory NK subset, by analogy with regulatory T cells [28]. In our study, patients showed lower frequencies of CD16^dim/neg^CD56^bright^ cells compared to controls (*p* = 0.027, Fig. 8), while no differences in percentages of other existing NK cell subtypes (i.e. CD16^bright^CD56^dim^, which is the major circulating subset, and two minor subsets of CD16^dim/neg^CD56^dim^ and CD16^bright^CD56^neg^ cells [29]) were revealed (data not shown). Correspondingly, the ratio of %CD56^dim^ NKs to %CD56^bright^ NKs displayed a tendency to increase in cervical cancer patients relative to the controls (significantly at *p* = 0.016 in stage IA patient group, Fig.8), presumably indicating a systemic change in the balance between effector and regulatory cells within the innate immune system promoted by invasive cervical cancer development. Along with Treg expansion, this change may contribute to systemic immune suppression in CIN/CC patients.

### Analysis of correlations: Putative mechanisms for the establishment of a suppressed immune status of cervical carcinoma patients

**Fig. 9:**
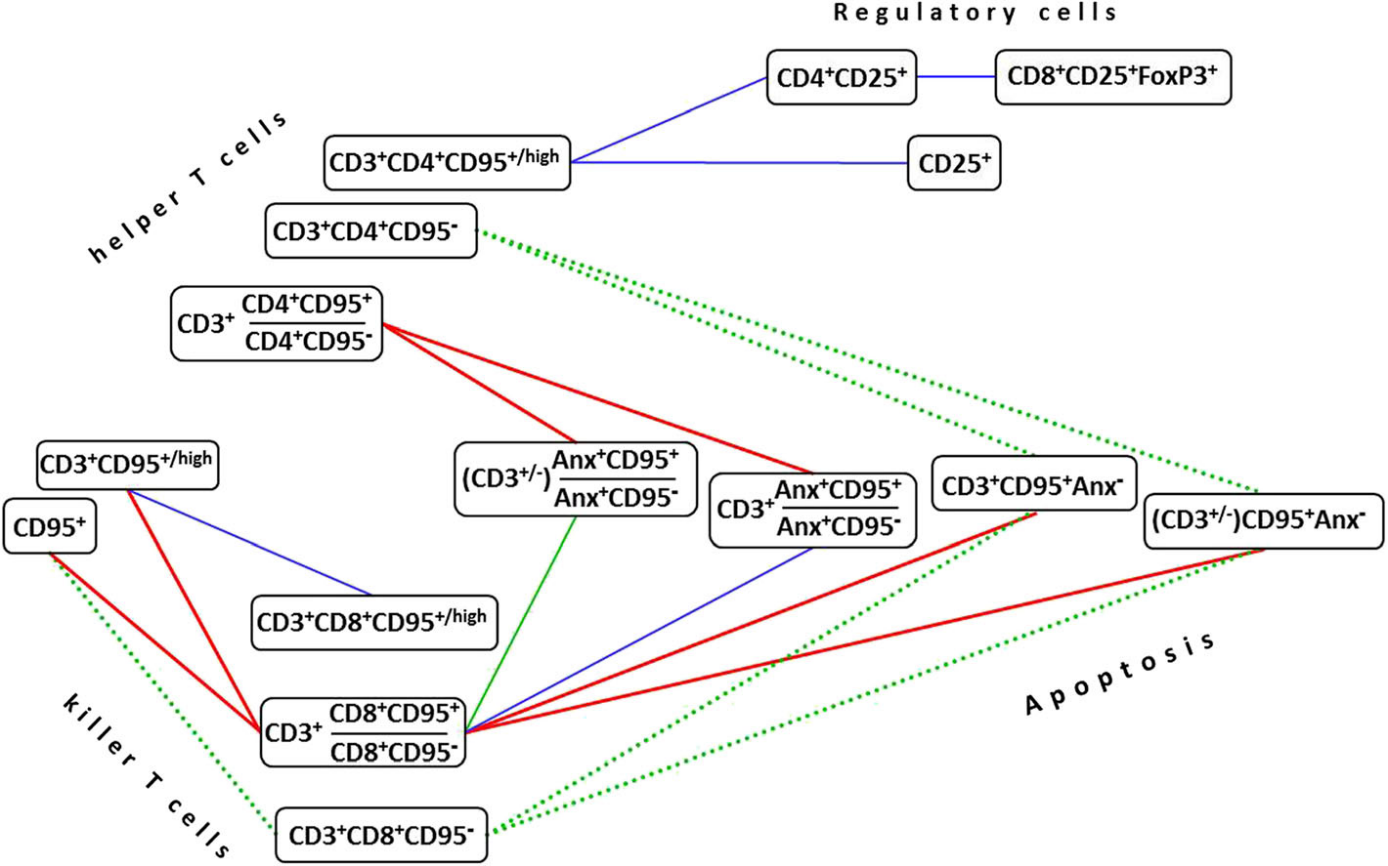
Correlations found between the percentages of different populations of circulating lymphocytes and their ratios in entire cohort of participants. The color of the lines corresponds to the strength of a relationship (the absolute value of the correlation coefficient): red for r ϵ[0.8; 0.9), green for r ϵ[0.7; 0.8), and blue for r ϵ[0.6; 0.7); *p* < 0.05. Dotted lines correspond to negative correlations between variables, solid lines show positive correlations. The phenotype (CD3^+/−^) refers to the total population of lymphocytes (including both CD3^+^ T cells and CD3^−^ non-T lymphocytes). Anx – Annexin V

**Fig. 10:**
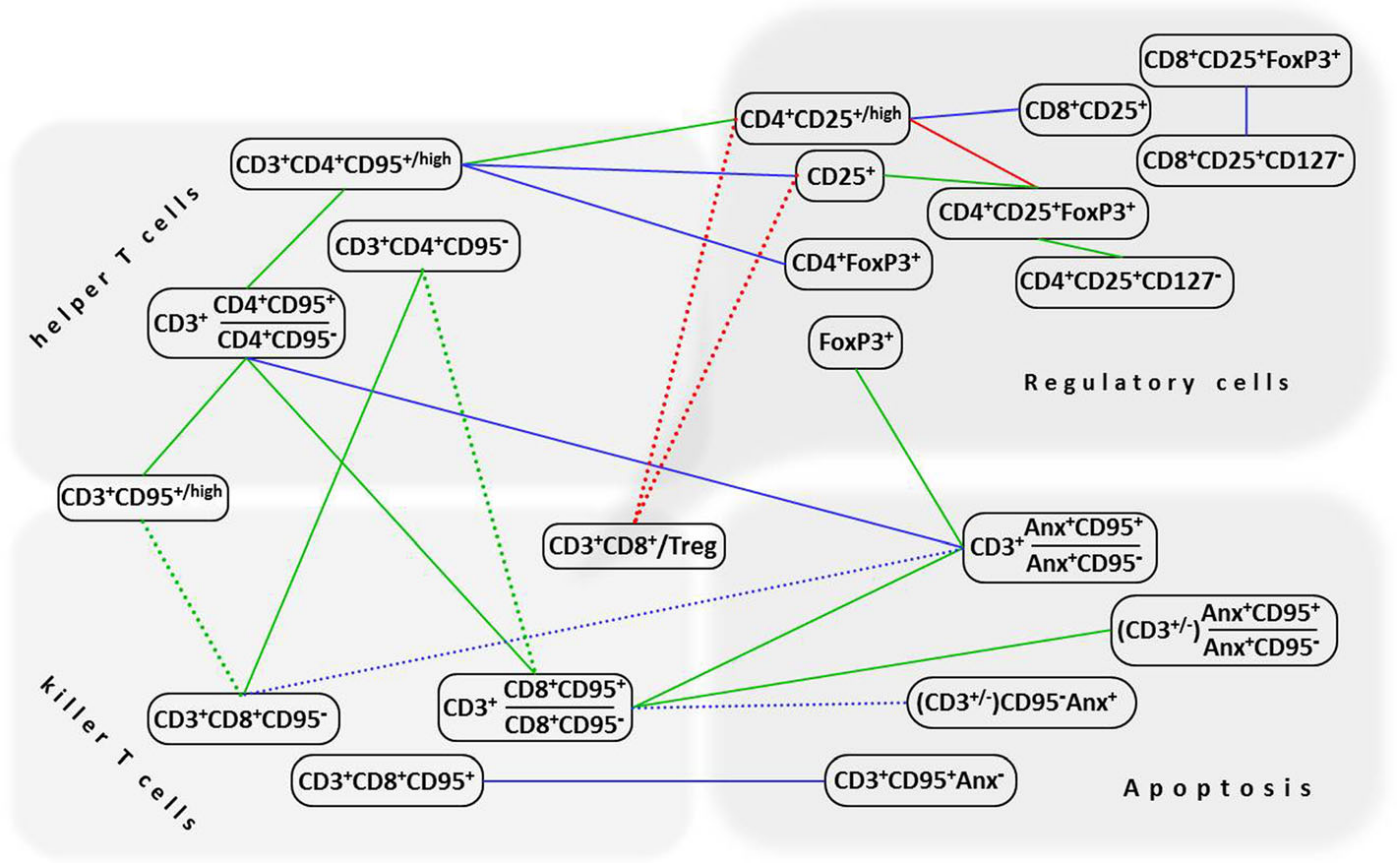
Correlations found between the percentages of different populations of circulating lymphocytes from patients with cervical pathology. The color of the lines corresponds to the strength of a relationship (the absolute value of the correlation coefficient): red for r ϵ[0.8; 0.9), green for r ϵ[0.7; 0.8), and blue for r ϵ[0.6; 0.7); *p* < 0.05. Dotted lines correspond to negative correlations between variables, solid lines show positive correlations. The phenotype (CD3^+/−^) refers to the total population of lymphocytes (including both CD3^+^ T cells and CD3^−^ non-T lymphocytes). Treg denotes the percentage of CD4^+^CD25^+^FoxP3^+^ cells; Anx – Annexin V

A total of 80 combinations of phenotypic parameters (*gates*) analyzed in the present study were nominally split into 3 functional categories (“Regulatory cells”, “CD95-expressing T cells”, and “Apoptosis”), and a search for correlations between them was performed on the basis of flow cytometric data. Most significant relationships (*r* > 0.6, *p* < 0.05) existing between the parameters (i.e. lymphocyte subpopulations) that were assigned to these functionally distinct categories are summarized in Figs.9 and 10. As follows from the results obtained in the whole cohort of participants (controls + patients), an increase in the percentage of CD95-positive CD4 or CD8 T cells relative to corresponding CD95-negative populations was correlated with the increased proportion of apoptotic (Anx^+^) lymphocytes co-expressing CD95, with this correlation being more pronounced in CD4^+^ Т cells, than in CD8^+^ T subset (Fig.9). Concomitantly, the lack of CD95 expression on CD4^+^/CD8^+^ T cells was associated with the absence of Annexin V binding in total T lymphocytes (as determined in independent probes). Interestingly, in contrast to CD4^+^ T-cells, the increase of CD95^+^ to CD95^−^ ratio in CD8^+^ Т cell population strongly correlated with the percentage of CD3^+^CD95^+^ Т lymphocytes that were negative for staining with Annexin V, as well as with the total amount (%) of CD3^+^CD95^+^ cells. In sum, the in vivo existence of these relationships could be accounted for based on the concept that upregulation of CD95/Fas receptor surface expression (resulting, for example, from lymphocyte activation in response to infection) represents a critical mechanism of induction of apoptosis in Т lymphocytes upon interaction with death ligands and, along with this, the observed change in CD95 may have distinct functional significance for CD4^+^ and CD8^+^ T cell subsets. In addition to these results, moderately strong positive correlation was revealed between the frequencies of cells expressing Treg-associated phenotype and CD4^+^CD95^+^ T lymphocytes (Fig. 9).

We next hypothesized that coordinated changes in the frequencies of the different populations of lymphocytes upon disease development may imply involvement of these populations in a common mechanism exploited by cervical cancer to establish immunosuppression. Therefore similar correlation analysis was done separately for the group of healthy women and the group of women with cervical neoplasms (CIN3/cancer in situ/ CC stage IA) in order to find out which of the relationships emerge (or become significantly stronger) in cancer, but are absent (or negligibly weak, *r* < 0.6) in normal controls. The scheme in Fig. 10 shows correlations between the proportions of lymphocyte populations that were found to have an absolute value >0.6 in the patient group (at *p* < 0.05), but <0.6 in the control group. In comparison with the results obtained using the full sample, CIN/CC patients exhibited stronger correlation between the expression of CD95 on CD4^+^ T cells and the frequency of regulatory CD4^+^CD25^+/high^ and CD4^+^FoxP3^+^ lymphocytes. The data also showed a strong negative correlation between the frequency of CD4^+^CD25^+/high^ cells (as well as overall expression of CD25 marker in circulating lymphocytes) and CD3^+^CD8^+^/Treg ratio in women with a diagnosis of CIN3/cancer in situ/ CC stage IA, but not in healthy donors. Additionally, the lymphocyte expression of FoxP3 factor and the level of CD95-associated apoptosis in T cells (estimated as the proportion of CD3^+^CD95^+^Anx^+^ lymphocytes) were correlated only in patient group (Fig. 10).

When considering the relation between the level of apoptosis and differential expression of CD95 in CD4^+^ and CD8^+^ T cells, one can notice that it is within the CD8^+^ T cell subset where the correlation between the relative amount of CD95-expressing cells (CD3^+^CD8^+^[CD95^+^/CD95^−^] ratio) and the level of Anx binding with CD95^+^ lymphocytes was found in the CIN/CC patient group only, whereas for CD4^+^ T lymphocytes this correlation was detectable in the control group as well (*r* > 0.7). Likewise, a correlation between elevated percentage of total CD95-expressing T lymphocytes (%CD3^+^CD95^+/high^) and elevated proportion of CD95-expressing cells within CD4^+^ subset (CD3^+^CD4^+^[CD95^+^/CD95^−^] ratio) was observed in patients (in the case of CD8^+^ T-cell subset, the correlation between these parameters had *r* > 0.6 both in patients and controls).

**Fig. 11:**
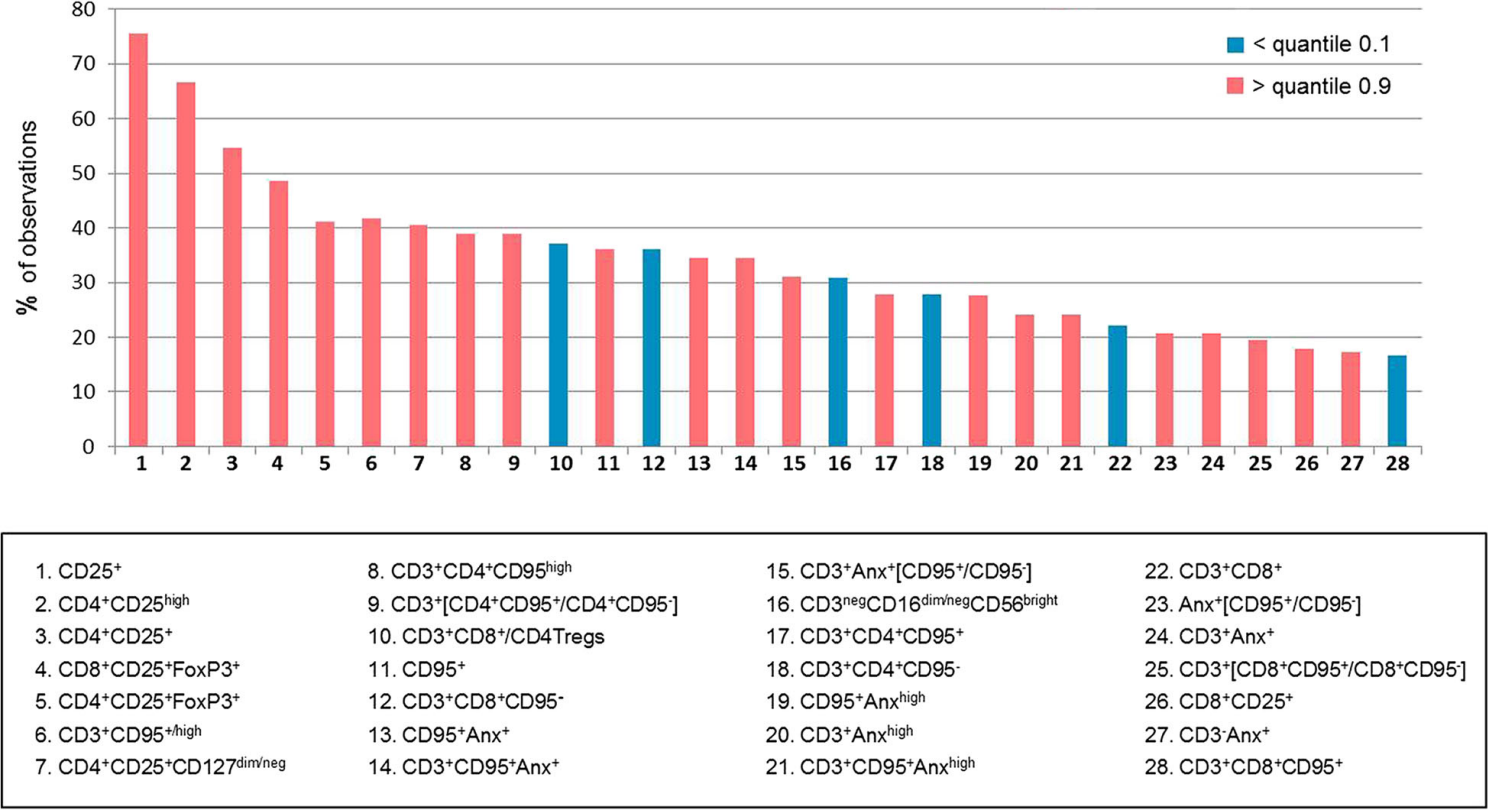
Histogram showing the proportion of observations for each phenotypic parameter (i.e. the frequencies of circulating lymphocyte populations with indicated phenotypes) in the patient group (plotted in decreasing order) that lie outside the ‘normal’ range which limits were estimated experimentally as the 0.1 and 0.9 quantiles for the control group of healthy donors; the percentage of values appeared to be less than the 0.1 quantile for parameters that exhibited decreasing trend in the patient group are shown in blue, the percentage of values that were greater than the 0.9 quantile value for variables with increasing trend in cancer patients are shown in red

For each phenotypic parameter measured by flow cytometry, we also analyzed the empirical distribution and determined 0.1 and 0.9 quantiles based on the values obtained for control group. The [0.1; 0.9] interquantile interval was taken as the ‘normal’ range and the number of values lying outside this interval in the patient group was divided to the total number of observations to obtain the proportion of values that differ from the normal range (Fig. 11). Reasoning from the results, we may hypothesize that Tregs may be one of the primary sources for other changes in cellular immune parameters, as more than 40–50% of patients with CIN3 or initial stages of CC progression (0-IA1) showed higher frequencies of Treg cells (both within CD4 and CD8 subset) in circulation in comparison with the ‘normal’ range. About one third of cases exhibited altered CD95 expression on T cells (above the ‘norm’ for the CD4^+^ subset and below for CD8^+^) and increased levels of Annexin V binding (Fig. 11). Taken together, women with early neoplastic lesions of the cervix, such as carcinoma in situ and microinvasive carcinoma, displayed a coordinated increase in expression of markers of regulatory T cells in blood CD4^+^/CD8^+^ lymphocytes, along with more pronounced cross-relationships between Treg numbers, CD95 expression on CD4^+^/CD8^+^ T cells, and apoptosis, with these interrelated changes being suggestive of the mechanisms responsible for the development of systemic immunological deficiency throughout cervical cancer progression.

## Discussion

Among known populations of suppressor cells, CD4 Tregs and their functional role in carcinogenesis are the most extensively studied; for example in the case of cervical cancer and its precursor lesions, a substantial body of research has convincingly demonstrated accumulation of CD4 Tregs in the tumor site. Based on these studies, mechanisms through which CD4^+^ cells can undergo differentiation into Tregs under the influence of tumor microenvironment, with assistance of dendritic cells and the stromal component, have been proposed. Fewer data are available on whether these processes can spread to systemic level; nevertheless, importantly, most investigators point to the fact that the increase in the level of peripheral blood CD4 Tregs is an early event in cervical cancer development, occurring at the stage of CIN2–3 [15, 16, 30], and is probably linked with the persistence of high-risk HPV infection [14]. Our data showing increased numbers of lymphocytes co-expressing Treg markers in the circulation of women with preinvasive and microinvasive cancer are in agreement with these findings. Therefore, one can expect pathological alterations that reflect the functioning of immunosuppressive mechanisms triggered by expansion of CD4 Treg population would also be detectable in the peripheral circulation during early stages of cervical cancer progression. An example to support this assumption is the study by Visser and co-authors who employed ex vivo system to demonstrate that CD4^+^CD25^high^ Tregs can inhibit IFNγ production by stimulated HPV16 E6/E7-specific Т cells isolated from peripheral blood of CIN and cervical cancer patients [15]; nonetheless the spectrum of relationships between CD4^+^Tregs and their potential target cell populations formed upon CIN/CC development remains largely uncharacterized.

Apart from inhibiting the functional activity of effector cells, ability to induce lymphocyte apoptosis was recognized as another crucial mechanism whereby tumors can antagonize antitumor immune responses. Through upregulation of CD95L/FasL, peripheral blood CD4 Tregs were also able to trigger apoptotic CD95/Fas-dependent death of autologous CD8^+^ Т effectors in a co-culture system, as was demonstrated in head and neck squamous cell carcinoma [2]. Accordingly, if helper/cytotoxic T lymphocytes upregulate their CD95 membrane expression, then expansion of Tregs in blood of cancer patients might result in enhanced levels of spontaneous apoptosis in T cells. Such relationships have been confirmed in some types of human cancer: for example, a coordinated increase in the number of CD95L^+^ Tregs and the level of Annexin V binding with CD95^+^ cytotoxic Т lymphocytes has been reported in the peripheral blood of hepatocellular carcinoma patients [5]. Another study published by Hoffmann and co-authors revealed significantly elevated frequencies of circulating CD3^+^CD95^+^ T cells that also displayed a higher level of Annexin binding in head and neck cancer (accounting for up to 100% in advanced disease), but a correlation between these markers of apoptosis and Treg numbers was not analyzed [4]. According to our findings, an increase in CD95 expression occurs in peripheral blood T cells in early-stage CC, which may represent a consequence of long-term exposure of T lymphocytes to virus-specific or/and tumor-associated antigens and may also reflect systemic activation status of the immune system over the course of HPV infection and progression of neoplasia. In one third of CC patients examined, the proportion of CD3^+^CD95^+^ cells stained positively for Annexin V exceeded the range of values defined for the healthy control group, suggesting that an elevated level of T-cell apoptosis is likely due to activation of CD95-dependent pathway. Notably, a strong association between the proportions of CD3^+^Anx^+^CD95^+^ cells and FoxP3^+^ lymphocytes was revealed in the CIN/CC patient group. Although the existence of such correlation cannot be appreciated as direct evidence of causal relationships, it nevertheless underpins the possibility that Tregs may execute their suppressive function through activation of T-cell apoptosis in preclinical stages of cervical carcinoma.

From the analysis of data on differential changes in the expression of CD95 and the level of apoptosis in CD4 and CD8 subsets of peripheral blood T cells available from published sources, it can be inferred that these changes exhibit different patterns depending on the type of cancer. For example, Xu and colleagues reported reduced expression of CD95 (as well as some other activation- and cytotoxicity-related markers) by circulating CD3^+^CD8^+^Т lymphocytes from patients with advanced lung cancer and proposed this reduction might be linked to impairment of killer T-cell function [31]. On the contrary, patients with gastric [32] and breast cancer [33] showed up-regulation of CD95 expression in peripheral blood CD8 Т cells, while head and neck cancer patients displayed increased numbers of CD95^+^ cells in both CD4 T helper and CD8 T killer subsets [4]. In blood samples from patients with preinvasive and microinvasive cervical cancer tested our study, we could observe significantly higher proportion of CD4^+^CD95^+^ Т lymphocytes with a concomitant tendency for decreased percentage of CD8^+^CD95^+^ Т lymphocytes compared to healthy controls. Since this decrease was not accompanied by an increase in the amount of CD8^+^CD95^−^ T cells, a plausible explanation for this observation may be the higher rate of apoptosis in CD8^+^ Т lymphocytes resulting in the more rapid elimination. Indeed, as follows from most studies, in which apoptosis-related markers were measured in CD4^+^ and CD8^+^ subsets of circulating T cells of cancer patients, CD8^+^ lymphocytes are more susceptible to apoptosis [2, 4, 32]. Although in the present study we did not compare the level of Annexin V binding between CD4 and CD8 T lymphocytes, we can speculate that the same feature could be responsible for the observed in CIN/CC group correlation between the relative abundance of Anx^+^CD95^+^ T cells and the proportion of CD8^+^CD95^+^ T cells, which appeared to be stronger than between Anx^+^CD95^+^ and CD4^+^CD95^+^ T cells. Regarding CD4+ effector T cells, other, CD95-independent, pathways mediated by Tregs to negatively regulate their activity are supposed to be more important [2], including those mediated by TGFβ, a cytokine secreted both by Tregs and cervical cancer cells [34] (as indicated above, female patients involved in our study had higher level of serum TGFβ than the controls). Interestingly, in CIN/CC patient group, a strong association was seen between the frequencies of CD4^+^CD95^+^ T cells and CD4^+^CD25^high^ cells (along with a more moderate correlation between CD4^+^CD95^+^ cells and CD4^+^FoxP3^+^ cells, Fig.10) suggesting that these cell populations may be involved in a common mechanism, whereas no such relationship was detected for CD8^+^CD95^+^ T cells.

Recent research on the mechanisms of immunosuppression employed by a developing tumor has provided novel insights on the diversity and function of other cell subsets (besides CD4 Tregs) capable of repressing effector activities of the immune system and thereby facilitating tumor evasion of immune surveillance. These include a relatively poorly studied population of CD8^+^Treg lymphocytes, whose role in tumor development and progression is not yet fully understood. There are only few published data on gynecological cancers, for example a significantly higher frequency of circulating CD8 Tregs has been described in patients with advanced ovarian carcinoma (stage III-IV) compared to patients with early-stage (I-II) or benign tumors of the ovary [35]. In the case of cervical cancer, there is one study by Chen and colleagues who revealed increased levels of circulating CD8^+^CD25^+^CD127^dim/neg^FoxP3^+^ lymphocytes in women with invasive cervical carcinoma (stage IA-IIB) and CIN3 compared to healthy counterparts, with no significant difference between CIN3 and invasive cancer detected [16]. Results from our study on CIN3 and microcarcinoma presented here are consistent with these observations. Also, several research teams have demonstrated that in spite of the fact that the population of CD8 Tregs is much less numerous in the peripheral circulation than CD4 Tregs, in in vitro assays they are nevertheless able to suppress T-cell proliferation (as effectively as, or even better than, CD4 Tregs), acting through the release of TGFβ or via other mechanisms [25, 35, 36]. Given that in experimental settings CD8^+^ T cells were shown to convert to suppressor CD8 Tregs under the influence of the tumor cell microenvironment [35, 37], it can be assumed that expansion of CD8 Tregs observed in systemic circulation of cervical cancer patients at a pre-metastatic stage might reflect important local level changes in their quantities, that provide a foothold for further tumor dissemination. This assumption was underpinned by Battaglia and co-authors [10] and Heeren and co-authors [11], who showed that CD8^+^FoxP3^+^ Т cells were more frequent in metastatic tumor-draining lymph nodes in early-stage cervical cancer. Apart from oncological diseases, CD8 Tregs are thought to play an important role in establishment of chronic viral infection, including herpesviruses (EBV, CMV), HCV and HIV (discussed in [38]). It is also possible that HPV engages CD8 Tregs for maintaining its long-term persistence, which may underlie an increase in the frequency of CD8 Tregs observed at earliest stages of CC progression.

Diverse types of cells endowed with regulatory properties have been discovered in the innate immune system; among these, NKregs have recently emerged as an important subpopulation whose functions and tissue distribution in different pathological conditions (including cancer) are presently attracting increasing interest. Owing to their high capacity to produce proinflammatory cytokines, NKregs are no longer regarded simply as a minor fraction of peripheral blood NK cells, but there are only a few types of cancer, for which their contribution to antitumor immunity have been described so far. We observed a decrease in the frequency of circulating CD56^bright^ NK lymphocytes in women diagnosed with cervical cancer, although the overall percentage of a major population of NK cells (CD56^dim^) didn’t differ significantly between patients and controls. Similar findings have been made in two other studies on head and neck cancer [39] and prostate cancer [6], whereas conflicting data have been reported for breast cancer (while Bauernhofer and co-authors [40] showed decreased levels of CD56^bright^ NK cells, Nieto-Velázquez and co-authors [41] revealed no changes in CD56^bright^ NK-cell numbers relative to healthy individuals). As a consequence, we were able to reveal an increased CD56^dim^/CD56^bright^ NK-cell ratio in the peripheral blood of cervical cancer patients with no difference between patients and controls in the total percentage of NK cells. Similar results have been reported by Bauernhofer et al. [40] and Koo et al. [6]. The most likely explanation for these data is that circulating CD56^bright^ NKregs are recruited to the lymphoid tissue (regional lymph nodes) and primary tumor site, where they comprise a significant portion of NK lymphocytes and are thought to serve as precursors for cytotoxic/effector CD56^dim^ NK cells [6]. While the distribution of CD56^bright^ cells between different tissue compartments in cervical cancer patients remains unclear, flow cytometry analysis of enzymatically dissociated samples of primary high-grade serous ovarian carcinoma confirmed that CD56^bright^ NK cells were indeed predominant among intratumoral NK lymphocytes [42].

In the present study, we focused on identifying phenotypic changes in circulating lymphocytes of patients with preinvasive and microinvasive carcinoma, since these disease stages represent, on the one hand, the ultimate result of malignant transformation, and, on the other hand, the transition from intraepithelial development to invasive growth, which could be considered as a turning point in tumor progression. In relation to histopathologic and clinical features, preinvasive and microinvasive lesions are very close, that is apparently why we didn’t observe significant differences in most parameters tested between respective subgroups of patients. However, the data obtained indicated that immunological defects seen in the periphery may accumulate during further CC progression, and additional studies that would include more advanced disease stages are needed to clarify this question. In this regard, some valuable results of a flow cytometric study of cell suspensions prepared from tumor-draining lymph nodes of female patients with stage IB1 cervical cancer [11, 12] are worth mentioning: in this study, the authors found altered proportions of CD4^+^ and CD8^+^ Т cells, along with altered expression of T-cell activation and immune checkpoint markers, increased frequencies of CD4^+^ and CD8^+^ Tregs, as well as a decreased CD3^+^CD8^+^/Treg ratio.

It is worth noting, in conclusion, that numerous studies now carried out in the field of cancer immunotherapy and aimed at developing new approaches to treat cancer, such as the use of therapeutic vaccines, targeted drugs (with primary attention to immune checkpoint inhibitors), and cytokine-based therapy, presuppose deep understanding of the processes that underlie both local and systemic dysfunctions of antitumor immunity in different kinds of malignancies and at different stages of disease progression. Immune regulatory networks that maintain cancer onset and progression (including the association between regulatory lymphocytes and the level of T cell apoptosis analyzed in the present work) may serve either as the source of molecular and cellular therapeutic targets or markers for monitoring the efficacy of immunotherapy and its combination with chemo−/radio−/targeted therapy regimens. For example, a vast amount of data has been amassed presenting opportunities for the use of CD4 Tregs as direct targets for new targeted agents with consideration of the features of Treg biology in different human cancers [43]. We have also demonstrated previously the informativity of some apoptosis-related markers in circulating lymphocytes for monitoring the effect of immunomodulatory treatment in patients with CIN3 and CC stage IA [27]. In view of the infectious etiology of cervical cancer, the most promising immune-based strategies for combination therapy of precursor lesions and invasive (especially metastatic) cancer are being discussed as a separate issue [44, 45]. It is plausible that some of these strategies may help disrupt pathologic interplay existing between CD4^+^/CD8^+^ Tregs and populations of cytotoxic lymphocytes and resulting in upregulation of apoptosis in the latter cells, which should be investigated further.

## Conclusions

Collectively, analysis of immunophenotypic data on peripheral blood lymphocytes from patients with CIN3/ carcinoma in situ or microinvasive carcinoma of the cervix suggests that the mechanisms, by which Tregs can expand their suppressive influence on antitumor immune responses and impair the balance between effector cell subsets (including via potentiation of CD95-mediated apoptosis), and which were previously established mostly with the use of murine models or other experimental systems, play a relevant role in promoting early cervical cancer progression and spread in humans. These pathological changes may also be accompanied by a regulatory/effector cell disbalance within non-T lymphocytes. Activation of the above mechanisms observed at the level of systemic circulation and coincident with the earliest lesions (virtually confined to epithelial layer) obviously reflects the existence of a complex cross-talk and interdependence between local immune reactions and responses of peripheral blood lymphocytes.

## Abbreviations

CC: Cervical cancer
CD: Cluster of differentiation
CIN: Cervical intraepithelial neoplasia
FSC: Forward scatter
HPV: Human papillomavirus
NK: Natural killer cells
NKreg: Regulatory NK cells
SSC: Side scatter
Treg: Regulatory T cells
